# Socioeconomic inequities in prostate cancer care: private *versus* public treatment settings pose a significant impact on overall survival

**DOI:** 10.31744/einstein_journal/2025AO0851

**Published:** 2025-04-17

**Authors:** Amanda Caroline de Souza Costa, Fernando Korkes, Jose Augusto Rinck, Frederico Timóteo Silva Cunha, Luciana Holtz de Camargo Barros, Stênio de Cássio Zequi, Maria Nirvana da Cruz Formiga

**Affiliations:** 1 AC Camargo Cancer Center São Paulo SP Brazil AC Camargo Cancer Center, São Paulo, SP, Brazil.; 2 Centro Universitário FMABC Santo André SP Brazil Centro Universitário FMABC, Santo André, SP, Brazil.; 3 Hospital Israelita Albert Einstein São Paulo SP Brazil Hospital Israelita Albert Einstein, São Paulo, SP, Brazil.; 4 Instituto Oncoguia São Paulo SP Brazil Instituto Oncoguia, São Paulo, SP, Brazil.

**Keywords:** Health services, Prostatic neoplasms, Neoplasm metastasis, Race factors, Social determinants of health, Health systems, Survival analysis, Public sector, Private sector, Brazil

## Abstract

**Objective:**

To evaluate the impact of assistance from public and private health systems on the overall survival of patients with metastatic prostate cancer.

**Methods:**

A total of 213 patients with metastatic prostate cancer treated at the A.C. Camargo Cancer Center, either in the Unified Health System or a private system, from January 2014 to December 2018 were analyzed. Multivariate analysis of overall survival was performed to adjust for the type of health assistance and other clinical prognostic factors.

**Results:**

Of 213 included patients, 147 (69%) were from the private system and 66 (31%) were from the Unified Health System. There was a significant difference in the median age at diagnosis between the systems (63.4 years for patients in the private system versus 67.2 years for patients in the Unified Health System, p=0.027). No significant differences in performance status were observed between the groups (p=0.695). Patients treated in the public system had access to fewer treatment lines (2.59 lines in the public system versus 3.04 lines in the private system, p=0.024). Our data revealed a longer median survival for patients with private health care (115 months for patients with private health care versus 78 months for patients in the Unified Health System, p=0.009). Multivariate analysis revealed that patients in the public system had a 66% higher risk of death than those in the private system.

**Conclusion:**

Our data demonstrate that patients in the Unified Health System have access to fewer lines of therapy for metastatic prostate cancer, resulting in shorter median survival than patients treated in the private system.

## In Brief

This study demonstrates that patients with metastatic prostate cancer treated in a public setting in Brazil had access to fewer lines of therapy, resulting in a shorter median survival than patients treated in the private system.

## Highlights

■ Men with metastatic prostate cancer in the public system have access to fewer treatment lines.
■ Survival in the public system was 3 years shorter than that in the private setting (78 versus 115 months).

## Graphical abstract

**Figure:**
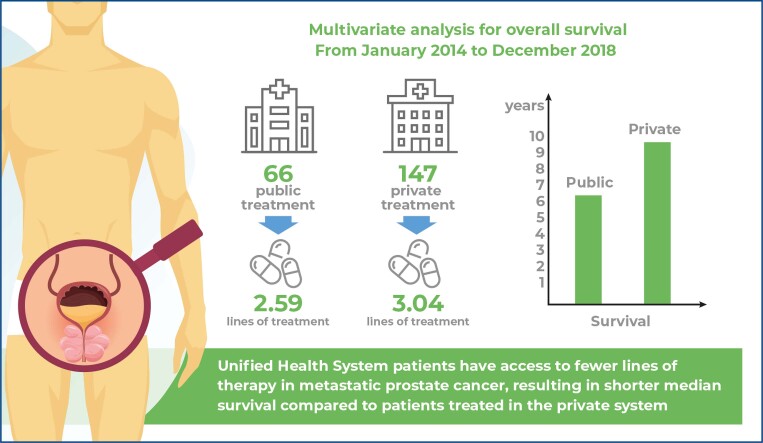

## INTRODUCTION

Prostate cancer (PCa) is a common disease that affects more than 70,000 Brazilians every year.^1^ Metastatic PCa (mPCa) includes androgen deprivation therapy (ADT), which has been used for more than 70 years. The combination of docetaxel with ADT, the hallmark chemotherapy for treating mPCa, was introduced only in the 2000s and it led to an improvement in symptoms and overall survival (OS) compared with the old cytotoxic scheme of mitoxantrone and ADT.^2^ Other drugs have been tested for mPCa after neoplastic cells developed androgen independence (castration resistance), such as new chemotherapeutic agents (cabazitaxel) and androgen-signaling-targeted agents (abiraterone or enzalutamide). Targeted radionuclide therapies, such as radium-223 dichloride, and most recently, 177Lu-PSMA radioligand therapy and poly ADP ribose polymerase (PARP) inhibitors have also been developed.^3,4^

In the last decade, various drugs, some of which have already been approved for the castration-resistant phase of mPCa and other new molecules, have been evaluated in earlier stages of mPCa (castration-sensitive and non-metastatic castration-resistant). These include new hormonal agents (apalutamide and darolutamide). Also, triplet therapy with ADT, docetaxel, and novel hormones has been evaluated in earlier stages of mPCa, leading to longer OS. Several studies have demonstrated that combining drugs in an earlier stage of mPCa leads to a longer OS. The mean OS of patients with mPCa has improved since the emergence of new drugs, new combinations of drugs, and drug sequencing strategies.^5-7^

Historically, the different outcomes in patients with PCa treated according to social and ethnic groups have been mainly attributed to biological differences and delays in diagnosis and treatment. Institutional racism has also been associated with disparities in healthcare access and treatment outcomes.^8,9^ In Brazil, the Public Health System (SUS *- Sistema* Ú*nico de Saúde*) is granted to all citizens. However, approximately 30% of the population also have private health insurance aimed at obtaining better and wider care. Most health institutions in Brazil that receive reimbursement from private insurance do not treat public patients and vice versa. However, the present study was performed at one of the few institutions that treat patients whose healthcare can be supported in both public and private settings. More than that, it means that these patients were treated at the same location and by the same professionals. The main difference is that all drugs granted to public patients are also granted to private patients. However, some drugs are granted only to privately owned patients.^10^

## OBJECTIVE

The present study evaluated the differences in the clinical characteristics of patients with metastatic prostate cancer treated under either the public or private health systems in a specialized Cancer Center in Brazil. We also explored potential factors that may influence survival outcomes in these two groups.

## METHODS

This unicentric descriptive retrospective cohort study included consecutive patients treated for mPCa between January 2014 and December 2018 at a single institution in São Paulo, Brazil. All patients were treated at the same institution by the same staff. The treatment protocols were the same; however, the availability of medication differed according to the payer.

Statistical analyses were performed using SPSS (SPSS for Mac OS X Corp., Armonk, NY, USA). Groups were compared with Pearson’s χ^2^ test. The Whitney test was used for continuous variables, and the Kaplan-Meier method and log-rank test were used for survival. Multivariate analysis of OS was performed to adjust for the type of health assistance and other clinical prognostic factors. Statistical significance was set at p<0.05. The Research Ethics Committee was approved by *Fundação Antônio Prudente A. A. C. Camargo Cancer Center*; CAAE: 12569219.6.0000.5432; # 3.996.416.

## RESULTS

A total of 1,374 men with PCa were treated between January 2014 and December 2018. Of these, 213 were diagnosed with mPCA, with 87 diagnosed with metastatic disease at their initial diagnosis. These patients were treated at our institution by either the public (n=147, 69%) or private system (n=66, 31%). The median age at diagnosis was 63.4 years for patients in the private system and 67.2 years for patients in the public system (p=0.027). Demographic data are shown in table 1.

**Table 1:** Patients’ demographics

	Private	Public	p value
	**n=147**	**n=66**	
Age (mean), years	64.3	67.2	0.027
Comorbidities, n (%)			
1-2	63 (75.9)	29 (82.9)	0.556
3 or more	20 (24.1)	6 (17.1)	0.556
ECOG, n (%)			
0-1	115 (80.4)	50 (76.9)	0.659
2-4	28 (19.6)	15 (23.1)	0.083
PSA (initial) – (median), ng/mL	93.25	18.6	0.083
PSA (metastasis) – (median), ng/mL	21.62	100.0	0.074
Metastasis, n (%)			
w/ bone metastasis	63 (42.9)	25 (37.9)	0.595
w/o bone metastasis	84 (57.1)	41 (62.1)	0.595
lymph nodes	62 (42.2)	32 (48.5)	0.479
w/ visceral metastasis	20 (13.6)	15 (22.7)	0.144
w/o visceral metastasis	127 (86.4)	51 (77.3)	0.144
Gleason score, n (%)			0.633
6	17 (12.9)	5 (8.2)	
7	28 (21.2)	14 (23.0)	
8-10	87 (65.9)	42 (68.9)	
Clinical stage at diagnosis, n (%)			<0.001*
I-III	70 (49)	12 (18.5)	
IV	73 (51)	53 (81.5)	
Treatment lines, n (%)			0.024*
0-2	48 (37.7)	33 (50.0)	
3-4	99 (67.3)	33 (50.0)	

*p<0.05.

ECOG: Eastern Cooperative Oncology Group; PSA: prostate specific antigen.

No differences in performance status were observed between the groups with ECOG 0-1 *versus* ECOG 2-4 scores (p=0.695). The number of comorbidities was similar between the groups (p=0.556), as was the Gleason score (p=0.633). The median PSA level at diagnosis was not significantly different in the public system (93.25 ng/mL) versus the private system (18.60ng/mL) (p=0.08). The median PSA levels at the diagnosis of metastasis was 100.0ng/mL *versus* 21.6 ng/mL in patients in the public versus private systems (p=0.074).

Exclusive bone metastasis occurred in 37.9% of patients in the public system *versus* 42.9% of patients in the private system (p=0.595). Lymph node metastasis was observed in 42.2% of patients in the public system versus 48.5% of patients in the private system (p=0.479). Visceral metastases occurred in 22.7% of patients in the public system *versus* 13.6% of patients in the private system (p=0.114).

Public patients had access to fewer treatment lines (2.59 lines) compared with the private system (3.04 lines) (p=0.024). While 67.3% of private patients received three or more lines of treatment, only 50% of public patients received three or more lines of treatment. The treatments are listed in table 2.

**Table 2:** The number of patients treated according to each line of therapy

	Private n=147	Public n=66	Total n=213
1^st^ line			
ADT	93 (63.27)	46 (69.70)	139 (65.26)
ADT + anti-androgen	23 (15.65)	8 (12.12)	31 (14.55)
Orchiectomy	5 (3.4)	1 (1.52)	6 (2.82)
ADT + abiraterone acetate	7 (4.76)	0	7 (3.28)
ADT + docetaxel	17 (11.56)	11 (16.67)	28 (13.15)
2^nd^ line			
ADT + anti-androgen	27 (18.37)	17 (25.76)	44 (20.66)
Diethylstilbestrol	1 (0.68)	0 (0.00)	1 (0.47)
Docetaxel	23 (15.65)	16 (24.24)	39 (18.1)
Abiraterone	30 (20.41)	0	30 (14.08)
Enzalutamide	23 (15.64)	0	23 (10)
Cabazitaxel	3 (2.04)	0	3 (1.41)
Paclitaxel	1 (0.68)	0	1 (0.47)
Oral cyclophosphamide	1 (0.68)	3 (4.55)	4 (1.88)
Anti-androgen	1 (0.7)	1 (1.52)	2 (0.94)
ADT	7 (4.76)	0	7 (3.29)
3^rd^ line			
Diethylstilbestrol	1 (0.7)	0	1 (0.47)
Docetaxel	32 (21.77)	14 (21.21)	46 (21.59)
Abiraterone	27 (18.37)	3 (4.55)	30 (14.08)
Enzalutamide	6 (4.08)	2 (3.03)	8 (3.76)
Cabazitaxel	9 (6.12)	0	9 (4.23)
Radium-223	5 (3.4)	0	5 (2.35)
Paclitaxel	0	2 (3.03)	2 (0.94)
Oral cyclophosphamide	2 (1.36)	4 (6.06)	6 (2.82)
4^th^ line			
Docetaxel	12 (8.16)	3 (4.55)	15 (7.04)
Abiraterone	10 (6.80)	0	10 (4.69)
Enzalutamide	13 (8.84)	0	13 (6.10)
Cabazitaxel	16 (10.88)	1 (1.52)	17 (7.98)
Radium-223	2 (1.36)	0	2 (0.94)
Paclitaxel	0	1 (1.52)	1 (0.47)
Oral cyclophosphamide	2 1.36)	3 (4.55)	5 (2.35)

ADT: androgen deprivation therapy.

**Figure 1:**
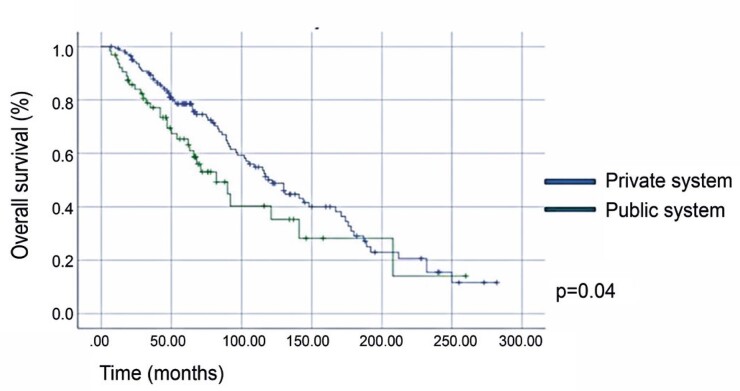
Comparison of the overall survival of patients treated at a public versus private health care system (median overall survival: 122 months for patients in the private system versus 82 months for patients in the public system, p=0.04)

Patients treated in a public setting had a significantly lower OS and died 37 months earlier (78 months *versus* 115 months for patients treated in the public *versus* private system, p=0.009). Multivariate analysis showed that patients with mPCa in the public system had a 66% higher risk of death than those in the private system. Figure 1 compares the OS data of patients treated in private *versus* public systems.

## DISCUSSION

The Brazilian SUS is responsible for treating 150 million citizens, with 60 million Brazilians receiving private insurance services. In this scenario, access to the public healthcare system in Brazil can be quite challenging, mainly because of socioeconomic disparities in the population, which may lead to considerable heterogeneity in diagnostic access across the continental country. Even in the European population, epidemiological studies have shown that privately insured patients surgically treated for PCa present with more favorable clinical and pathological characteristics, ultimately leading to lower disease recurrence and better oncological outcomes.^11,12^ Despite several efforts, such as federal law 12.732, which reinforces the oncological patient’s right to be treated for a maximum of 60 days after diagnosis to guarantee oncological patients’ quick access to treatment in the Brazilian public health system, SUS access remains a challenge to overcome. There are different regulations to access treatments and medications between the public and private systems. In the last decade, many medications have been incorporated into the treatment arsenal, resulting in significant improvements in survival. Although racial and social disparities are associated with barriers to the diagnosis and treatment of patients with PCa, we can compare access to drugs with mPCa outcomes in a few scenarios.

It is well known that the number of medications accessible to the public is lower in Brazil because drug incorporation takes a much longer time.^8^For the public system, drug incorporation is granted by the National Committee for Technology Incorporation in the Unified Health System (CONITEC - *Comissão Nacional de Incorporação de Tecnologias no SUS*) and regulated by the Health Ministry. After approval by the federal agency of health surveillance (ANVISA - *Agência Nacional de Vigilância Sanitária*), it normally takes several years for a drug to be incorporated and granted to patients. On the other hand, In the private setting, the National Supplementary Health Agency (ANS - *Agência Nacional de Saúde Suplementar*) regulates drug incorporation in a much more expedited fashion. Drug access is granted approximately 120 days after ANVISA approval according to federal law (MP 1.067/2021 and Lei 9.656, de 1998).

In the SUS, drugs available for mPCa include androgen deprivation agents, docetaxel, and, more recently, abiraterone. Although abiraterone has recently been approved (MS-SCTIE no. 38/19), most public institutions do not offer this drug because of financial limitations. New-generation antiandrogens, such as enzalutamide, darolutamide, and apalutamide, are not currently incorporated into the public setting. This is the same for drugs such as immune checkpoint inhibitors, PARP inhibitors, cabazitaxel, and theranostics. Some patients end up having the opportunity to receive further lines of treatment through clinical trials. Others receive more ancient and less effective cytotoxic drugs, such as mitoxantrone or cyclophosphamide. As expected, we found in this study that patients in the private setting received more lines of treatment (3.04 *versus* 2.59, p=0.024).

Our study had several important findings. First, our patients were treated at the same institution by the same medical staff, which reduced decision biases and protocol disparities. In this scenario, men treated in the public system had a significantly shorter OS. Access to private insurance resulted in an additional life expectancy of 37 months (115 *versus* 78 months, p=0.009). The odds of death after one, three, and five years were also higher for patients with public access to treatment. Previous studies have demonstrated disparities in whether patients have health insurance. For instance, for patients treated for breast, colon, lung, prostate, and bladder cancer in the USA, the risk of death within five years was significantly higher if they did not have health insurance (41-97%).^13^ The main difference from our study is that all of our patients had access to a health system, and even so, their life expectancy was different. In the present study, multivariate analysis demonstrated that worse OS was associated with stage IV disease at diagnosis (91% higher risk of death) and treatment in the SUS (66% higher risk of death).

Previous studies have demonstrated that diagnoses occur at later stages of disease with the Brazilian public system.^14,15^ In our study, we observed that the diagnosis of stage IV PCa was more common in the public system (81.5% *versus* 51%, p<0.001). PSA levels at diagnosis was higher for patients in the public system, although the difference was not statistical significance (93.25ng/mL *versus* 18.60ng/mL in the private setting, p=0.08). Although not statistically significant, the median PSA level at the diagnosis of metastasis was also higher in patients in the public system (100.0ng/mL) *versus* patients in the private system (21.6ng/mL) (p=0.074). Advanced stage at diagnosis has not only been attributed to health insurance conditions, but also to socioeconomic factors. A Swiss study demonstrated that patients with less favorable socioeconomic conditions had twice the mortality rate.^8^ In Brazil, this late diagnosis in lower-income patients has also been demonstrated in a previous study.^16^

A similar study published by our group several years ago demonstrated that the same difference was observed in patients with metastatic renal cell carcinoma. Patients treated in the SUS had significantly worse OS, possibly because of a poorer prognosis at presentation and less drug access.^10^

In recent years, many patients have received combined treatment immediately after the diagnosis of mPCa. Addition of abiraterone, enzalutamide, apalutamide, docetaxel, docetaxel, and darolutamide to ADT has increased survival rates. These combinations used early in the course of the disease seem to improve the OS more than when subsequently used.^6,17,18^

Our study had some limitations. First, its retrospective nature and small sample size limited further analysis. Furthermore, it is important to acknowledge that age and stage at diagnosis were not controlled, which may have affected our study outcomes. Additionally, some patients were referred to our institution after they had already received initial treatment. These patients were considered for analysis from the time of the first drug administration after the diagnosis of mPCa. Some patients were also included before the approval of new-generation antiandrogens and docetaxel in the hormone-sensitive mPCa setting. We also did not evaluate the patients as hormone-sensitive or castration-resistant. However, a real-life study can be performed at an institution with unique characteristics. This could demonstrate the frailty and limitations of primary care and access to treatment in public settings. Although these patients were treated at a specialized cancer center, their OS was significantly shorter because of these limitations in a public setting.

After decades of exclusive use of ADT for mPCa, several new drugs have been developed. These drugs have significantly improved the mean OS of patients through new drug combinations or drug sequencing strategies. Access to new cancer medications is a major global challenge throughout the world. However, these challenges are even greater in developing countries. Therefore, it seems reasonable to search for solutions that could grant the benefits of new technologies not only to the wealthiest, but also to those with less favorable socioeconomic conditions.

## CONCLUSION

Our data demonstrate that patients with metastatic prostate cancer treated in the public system were diagnosed at a more advanced stage with higher PSA levels. They had access to fewer lines of therapy, reflecting an overall survival of almost four years shorter than that of patients treated in the private system. Despite other adverse conditions, the number of lines of treatment received was an independent risk factor for mortality.

## References

[1] Brasil, Ministério da Saúde, Instituto Nacional de Câncer José Alencar Gomes da Silva (2023).

[2] Tannock IF, de Wit R, Berry WR, Horti J, Pluzanska A, Chi KN, Oudard S, Théodore C, James ND, Turesson I, Rosenthal MA, Eisenberger MA, TAX 327 Investigators (2004). Docetaxel plus prednisone or mitoxantrone plus prednisone for advanced prostate cancer. N Engl J Med.

[3] Jia AY, Kiess AP, Li Q, Antonarakis ES (2023). Radiotheranostics in advanced prostate cancer: current and future directions. Prostate Cancer Prostatic Dis.

[4] Hussain M, Mateo J, Fizazi K, Saad F, Shore N, Sandhu S, Chi KN, Sartor O, Agarwal N, Olmos D, Thiery-Vuillemin A, Twardowski P, Roubaud G, Özgüroglu M, Kang J, Burgents J, Gresty C, Corcoran C, Adelman CA, de Bono J, PROfound Trial Investigators (2020). Survival with Olaparib in Metastatic Castration-Resistant Prostate Cancer. N Engl J Med.

[5] Fizazi K, Foulon S, Carles J, Roubaud G, McDermott R, Fléchon A, Tombal B, Supiot S, Berthold D, Ronchin P, Kacso G, Gravis G, Calabro F, Berdah JF, Hasbini A, Silva M, Thiery-Vuillemin A, Latorzeff I, Mourey L, Laguerre B, Abadie-Lacourtoisie S, Martin E, El Kouri C, Escande A, Rosello A, Magne N, Schlurmann F, Priou F, Chand-Fouche ME, Freixa SV, Jamaluddin M, Rieger I, Bossi A, PEACE-1 investigators (2022). Abiraterone plus prednisone added to androgen deprivation therapy and docetaxel in de novo metastatic castration-sensitive prostate cancer (PEACE-1): a multicentre, open-label, randomised, phase 3 study with a 2 × 2 factorial design. Lancet.

[6] Smith MR, Hussain M, Saad F, Fizazi K, Sternberg CN, Crawford ED, Kopyltsov E, Park CH, Alekseev B, Montesa-Pino Á, Ye D, Parnis F, Cruz F, Tammela TLJ, Suzuki H, Utriainen T, Fu C, Uemura M, Méndez-Vidal MJ, Maughan BL, Joensuu H, Thiele S, Li R, Kuss I, Tombal B, ARASENS Trial Investigators (2022). Darolutamide and Survival in Metastatic, Hormone-Sensitive Prostate Cancer. N Engl J Med.

[7] Gillessen S, Armstrong A, Attard G, Beer TM, Beltran H, Bjartell A (2022). Management of Patients with Advanced Prostate Cancer: Report from the Advanced Prostate Cancer Consensus Conference 2021. Eur Urol.

[8] Rapiti E, Fioretta G, Schaffar R, Neyroud-Caspar I, Verkooijen HM, Schmidlin F (2009). Impact of socioeconomic status on prostate cancer diagnosis, treatment, and prognosis. Cancer.

[9] Lillard JW, Moses KA, Mahal BA, George DJ (2022). Racial disparities in Black men with prostate cancer: a literature review. Cancer.

[10] Leite LM, Bergerot PG, Dettino AL, Rinck JA, Zequi SC, Formiga MN (2021). Influence of treatment access on survival of metastatic renal cell carcinoma in brazilian cancer center. Int Braz J Urol.

[11] Briganti A, Blute ML, Eastham JH, Graefen M, Heidenreich A, Karnes JR (2009). Pelvic lymph node dissection in prostate cancer. Eur Urol.

[12] Gallina A, Karakiewicz PI, Chun FK, Briganti A, Graefen M, Montorsi F (2007). Health-insurance status is a determinant of the stage at presentation and of cancer control in European men treated with radical prostatectomy for clinically localized prostate cancer. BJU Int.

[13] Niu X, Roche LM, Pawlish KS, Henry KA (2013). Cancer survival disparities by health insurance status. Cancer Med.

[14] Maluf FC, Gillessen S (2021). Consensus on the Screening, Staging, Treatment, and Surveillance of Localized, Recurrent, and Metastatic Prostate Cancer: The First Global Prostate Cancer Consensus Conference for Developing Countries. JCO Glob Oncol.

[15] Korkes F, Smaidi K, Timoteo F, Glina S (2022). Recommendations for prostate cancer diagnosis and treatment during COVID-19 outbreak were not followed in Brazil. Int Braz J Urol.

[16] Nardi AC, Reis RB, Zequi SC, Nardozza A (2012). Comparison of the epidemiologic features and patterns of initial care for prostate cancer between public and private institutions: a survey by the Brazilian Society of Urology. Int Braz J Urol.

[17] Sweeney CJ, Chen YH, Carducci M, Liu G, Jarrard DF, Eisenberger M (2015). Chemohormonal Therapy in Metastatic Hormone-Sensitive Prostate Cancer. N Engl J Med.

[18] Small EJ (2017). Redefining Hormonal Therapy for Advanced Prostate Cancer: results from the LATITUDE and STAMPEDE Studies. Cancer Cell.

